# Impact of sanitation and socio-economy on groundwater fecal pollution and human health towards achieving sustainable development goals across India from ground-observations and satellite-derived nightlight

**DOI:** 10.1038/s41598-019-50875-w

**Published:** 2019-10-23

**Authors:** Abhijit Mukherjee, Srimanti Duttagupta, Siddhartha Chattopadhyay, Soumendra Nath Bhanja, Animesh Bhattacharya, Swagata Chakraborty, Soumyajit Sarkar, Tilottama Ghosh, Jayanta Bhattacharya, Sohini Sahu

**Affiliations:** 1 School of Environmental Science and Engineering, Indian Institute of Technology, Kharagpur, India; 2 Department of Geology and Geophysics, Indian Institute of Technology, Kharagpur, India; 3 Department of Humanities and Social Sciences, Indian Institute of Technology, Kharagpur, India; 4 Faculty of Science and Technology, Athabasca University, 1 University Dr., Athabasca, AB T9S 3A3 Canada; 5 Water and Sanitation Support Organization, Public Health Engineering Department, Govt. of West Bengal, Kolkata, India; 6 Cooperative Institute for Research in Environmental Sciences (CIRES), CU, Boulder, Earth Observation Group, NOAA National Centers for Environmental Information, Boulder, Colorado USA; 7 Department of Mining Engineering, Indian Institute of Technology, Kharagpur, India; 8 Department of Economic Sciences, Indian Institute of Technology, Kanpur, India

**Keywords:** Environmental impact, Hydrology

## Abstract

Globally, ~1 billion people, mostly residing in Africa and South Asia (e.g. India), still lack access to clean drinking water and sanitation. Resulting, unsafe disposal of fecal waste from open-defecation to nearby drinking water sources severely endanger public health. Until recently, India had a huge open-defecating population, leading declining public health from water-borne diseases like diarrhoea by ingesting polluted water, mostly sourced to groundwater. However, in recent past, sanitation development to achieve Sustainable Development Goals (SDGs) has been encouraged throughout India, but their effect to groundwater quality and human health conditions are yet-unquantified. Here, for the first time, using long term, high-spatial resolution measurements (>1.7 million) across India and analyses, we quantified that over the years, groundwater fecal coliform concentration (2002–2017, −2.56 ± 0.06%/year) and acute diarrheal cases (1990–2016, −3.05 ± 0.01%/year) have significantly reduced, potentially influenced by sanitation development (1990–2017, 2.63 ± 0.01%/year). Enhanced alleviation of groundwater quality and human health have been observed since 2014, with initiation of acceletated constructions of sanitation infrastructures through Clean India (Swachh Bharat) Mission. However, the goal of completely faecal-pollution free, clean drinking water is yet to be achieved. We also evaluated the suitability of using satellite-derived night-time light (NL_an_, 1992–2013, 4.26 ± 0.05%/year) as potential predictor for such economic development. We observed that in more than 80% of the study region, night-time light demonstrated to be a strong predictor for observed changes in groundwater quality, sanitation development and water-borne disease cases. While sanitation and economic development can improve public health, poor education level and improper human practices can strongly influence on water-borne diseases loads and thus health in parts of India.

## Introduction

“Access to safe water and sanitation, and sound management of freshwater ecosystems are essential to human health and to environmental sustainability and economic prosperity^1^”. Environmental pollution (including polluted water and improper sanitation) has been attributed to be the cause for 1/6^th^ of all pre-mature deaths in the world, in 2015, totaling to >9 million people, which are more deaths caused by war, hunger or any other wide-spread diseases taken together^2^. More than 90% of these deaths occur in the poor and developing countries of Asia and Africa, and the pollution-borne health conditions cost 6.2% of global economy^2^. Up to 1/3^rd^ of the present global population (i.e. >2 billion people) still lack access to improved water and sanitation, and predominantly live in the lower income, rural areas of sub-Saharan Africa and South-Southeast Asia^3^. Universal access to such basic human requirements would need urgent management of fecal waste and ending the unsafe practice of open defecation that causes severe risk to public health by exposure to fecal-pathogen polluted water^4^, unequally impacting the poor, women and children in developing countries^5^. Such crisis may only be addressed through overall development, and understanding the pathway of pathogens in hydrologic systems^6^. Thus, ~10% of the global human disease burden gets linked to improper sanitation-borne diseases and unsafe water, killing ~1.4 million children per year, more than malaria, measles, and AIDS combined^7–9^. In South Asia (specifically India), where ~23% of the global population lives on <4% of Earth’s surface, more people are now living in places with chances of fecal exposure^3^. While drinking water treatment is certainly very effective in reducing diarrhoea^10^, it’s imperative that less-contaminated water would provide better chances of human health alleviation. Over 117,000 Indian children, under age of five, die each year, from drinking water-borne diseases acute diarrhoeal diseases, with millions more affected with chronic enteric diseases^11^. Groundwater is the most prevalent potable water source across India^12^. However, studies on relationships between water-sanitation, public health and development have been very limited, yet, to investigate these relationships in large-scale^13^. The lack of consensus is related to the uncertainty on the causes of low sanitation coverage^14^.

Globally, between the years 2000 and 2015, only ~9% more people got access to improved safe water and basic sanitation, with ~1 billion people still defecating in the open. Among half of the population (>500 million) reside in the rural and peri-urban areas of India, and register the highest pollution-related deaths in the world^2^. As Indian economic development progressed during the last two decades, public health was expected to improve substantially, specifically as a result of promotion of policies from 2014, to eradicate open-defecation and access to safe water and proper sanitation for all residents (~1.3 billion) by 2019 through “Clean India (Swachh Bharat) Mission^15^”. Local-scale studies described these efforts to be “infrastructure-centered” and “supply-led” and have only moderate effect^14,16,17^. Notwithstanding these efforts and observations, it is unclear how these interventions would eventually influence the water-borne pathogens in drinking water and improving public health, over a long time. Quantifications of the outcome of such wide-scale interventions by improving drinking water to reduce diseases burden are not that common. Moreover, questions remain regarding how traditional practices and perception of purity, pollution and social structures may influence or interfere with proper sanitation and ending open defecation^17^.

In this study, we delineate the spatio-temporal trends of microbial water quality in groundwater reflected by groundwater-borne fecal coliform concentrations (**FC**, 2002–2017) and water-borne diseases, represented as acute diarrhoeal cases (**AD**, 1990–2016) across major parts of Indian region, as a consequence of household sanitation (**SAN**, 1990–2017). We also used satellite-based, night-time light, a widely used indicator of urbanization (**NL**, 1992–2013), to analyze it’s use as a predictor for changes in water quality and related health condition trends. Also, this helped us to evaluate the effect of changing land-use and urbanization on evolving water quality.

## Results and Discussion

**Figure 1:**
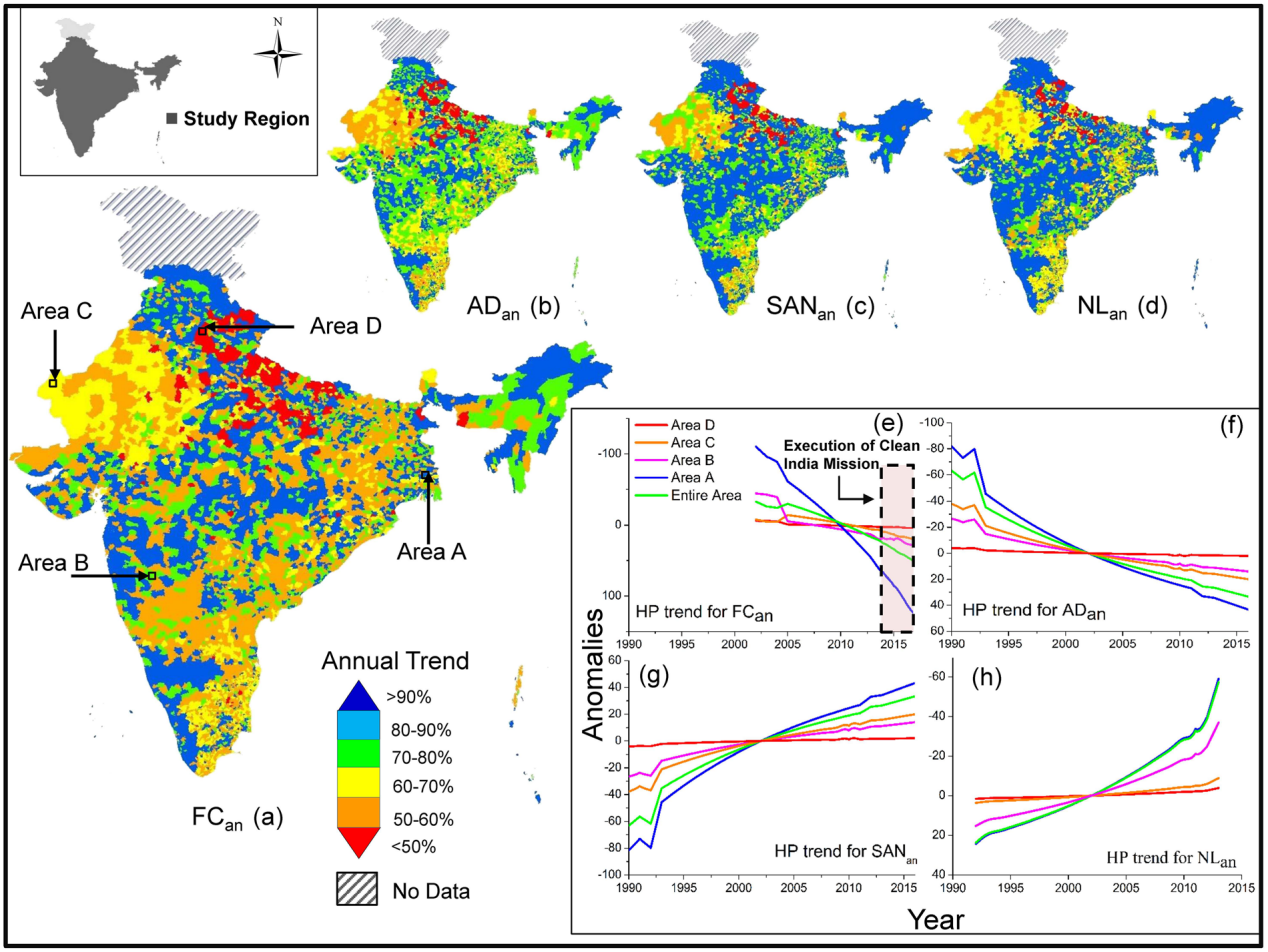
Map of study area across India, showing (**a**) annual linear trend of groundwater fecal coliform anomaly (FC_an_, 2002–2017) in each of the administrative blocks or equivalents (BLKs, n = 7010 BLKs) within the study period across the study region (shown in the inset map on top left). Linear slope of FC_an_ for the entire study area is −2.56 ± 0.06%/year for the data period. The map also show locations of the detailed study areas A (located in Highly Improved, FC_an_ decrease >90% within the study period), B (Improved, >70–90%), C (Moderately Improved, >50–70%) and D (Less Improved, <50%); annual linear trends of anomalies of (**b**) acute diarrheal cases (AD_an_, 1990–2016, −2.93 ± 0.03%/year for entire study area), (**c**) household sanitation structures (SAN_an_, 1990–2017, 2.63 ± 0.06%/year) and (**d**) night-time light (NL_an_, 1992–2013, 4.26 ± 0.05%/year); (**e**–**h**) non-linear, Hordick Prescott (HP) trends of the entire and detailed study areas (A, B, C and D) for FC_an_, AD_an_, SAN_an_ and NL_an_.

We delineated the long-term, annual trends of microbial groundwater quality, as fecal coliform concentration, and related water-borne diseases e.g. Acute Diahorea in India (Fig. 1), from ground-based measurements (n = 1,726,233) collected from a maximum of 7010 Indian administrative blocks or block equivalents (BLK) across the study region (Fig. 1). A block is an Indian district sub-division, defined for the purpose of government land administrative purpose, and is considered as the smallest unit of the Indian administration division. FC has cumulatively decreased across the study region by 38.5% from 2002 (mean ~33 MPN/100 mL) to 2017 (~24 MPN/100 mL), although yet to achieve the goal of no-FC clean drinking water. However, there is a discernable spatial variability in trends of such improvement across the study region (Fig. 1), with the range of improvements varying between >90% to <50% within the study period. About 3000 BLKs showed >90% cumulative improvement for FC within the study period (*highly improved*), with another ~1600 BLKs having improvement between 70–90% (*improved*), and ~500 BLKs having 50–70% (*moderately improved*). However, ~1700 BLKs have <50% improvement (*less improved*). To study and quantify this FC spatial variability and causes, and resulting human health impacts, we selected four detailed study areas, across the study region (Fig. 1), each containing 30 BLKs. Each of these detailed study areas, correspond to one of the aforesaid areas with FC improvement, *highly improved* (detailed study Area A), *improved* (Area B), *moderately improved* (Area C) and *less improved* (Area D) areas (Fig. 1a). These detailed study areas were selected based on ground conditions and additional high-resolution data availability for detailed numerical and statistical analyses (please see Methods and SI), and also were used for up-scaling our observations and conclusions to other data-deficient areas across the study region.

We also found that AD has cumulatively decreased by 79.3% between 1990 (n ~202 million cases) and 2016 (n ~42 million cases) (Figs 1 and S1). Linear trends of FC anomalies (FC_an_) and AD anomalies (AD_an_) within the study period suggest changes at the rates of −2.56 ± 0.06%/year and −2.93 ± 0.03%/year; respectively (Fig. 1). The rates of changes were found to be increasing (i.e. water quality and health condition improving) since in the 2014 (inception of “Clean India Mission”), with 6.02%/year and 7.96%/year for FC_an_ and AD_an_, respectively.

**Figure 2:**
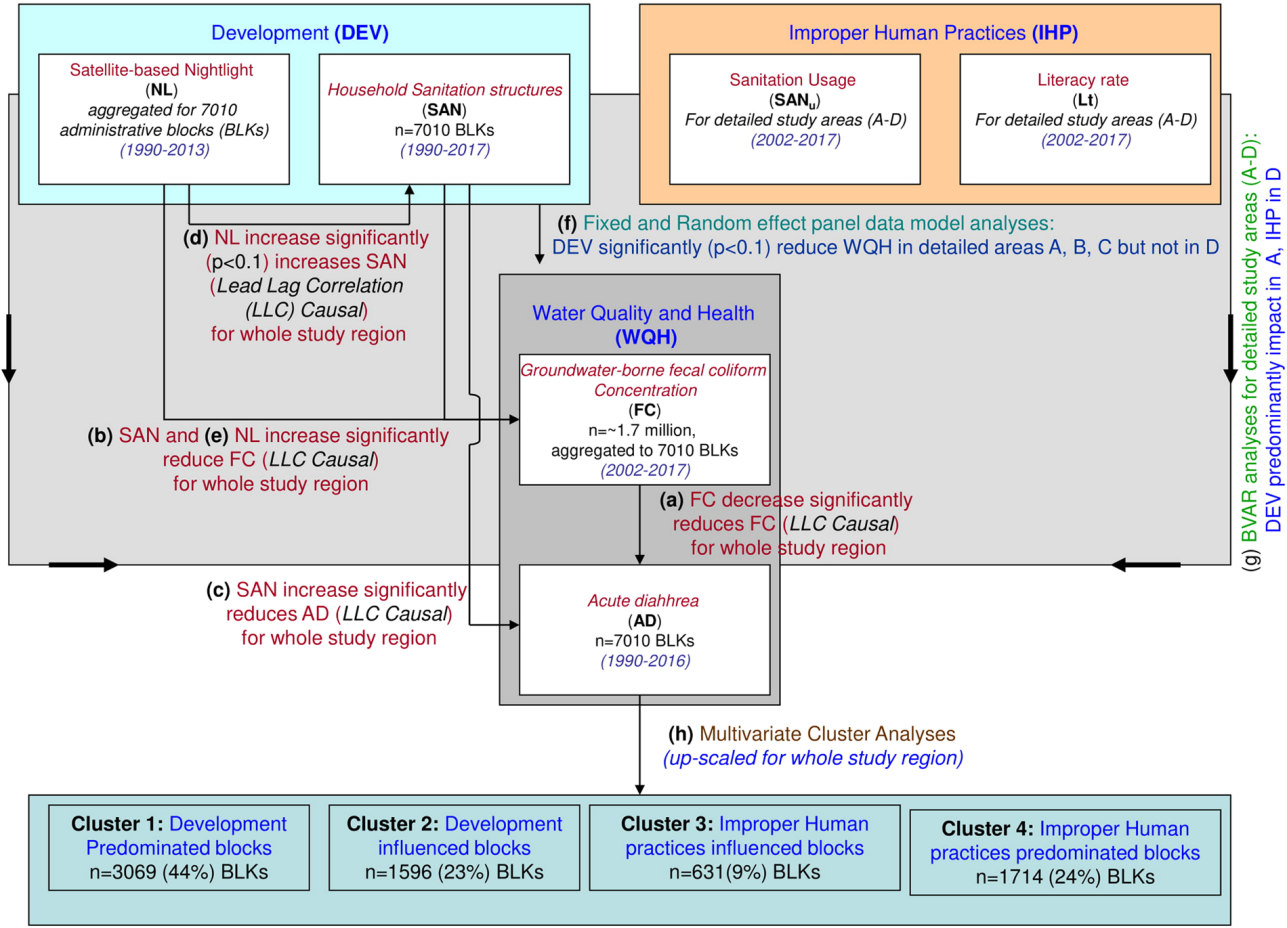
Schematic flowchart of data and methods applied in this study, displaying the parameters that have used e.g. FC, AD, SAN, NL, SAN_u_, Lt (white boxes) and analyzed relationships [(**a**) through (**h**)]. The parameters are listed with the numbers of observation and time period used in the study. The relationships also summarize the outcome of the analyses.

Analyses of linear trends in the detailed study area A demonstrates decrease in FC ~93% (FC_an_ linear trend: 10.91%/year) and AD ~91% (AD_an_ linear trend: 12.63%/year) for the study period. Similarly, B: ~84% (6.96% year), AD: ~82% (9.22%/year), C: ~66% (4.12%/year) and ~63% (7.61%/year), and D: ~34% (2.09%/year) and ~38% (2.33%/year) show discernable differences. Non-linear Hodrick-Prescott (HP) trends of FC_an_ and AD_an_ also indicate overall decrease (Fig. 1)^18^. FC_an_ shows significant strong positive correlation with AD_an_ (r = 0.99, p < 0.01) for the entire study area and for areas A (r = 0.99, p < 0.01), B (r = 0.95, p < 0.01) and C (r = 0.85, p < 0.01), but not in D (r = 0.72, p < 0.01) (Fig. 2).

While, FC and AD can be related, it is not necessary that all of the microbial pollution are sourced to FC, and will be directly influencing AD. Notwithstanding this observation, it was found that the lead-lag correlation (LLC) causality test suggests FC is strongly predicting AD for the entire study region, as well as detailed study areas A through D, both contemporaneously and in successive years^19^ (Fig. 2a, also see SI). These analyses suggest that, in general, the reduction of FC in groundwater is helping in alleviating the water-borne diseases like AD, across the study region, excluding areas with persistent lower improvement of FC (e.g. Area D or similar areas). The observed lower correlations of FC and AD in Area D, suggest that AD in these areas may also be caused by additional and/or unaccounted pathogen exposure risks, other than drinking groundwater pathways^20^. These results are in overall agreement with previous literature^21^ that improvement of water quality in south-east Asia has led to overall decrease in number of acute diarrhoeal cases related death due improvement of water quality.

In last couple of decades, the administrative authorities in India have promoted development of millions of household sanitation structures^22^, with enhanced promotion since 2014. We hypothesize that the aforesaid changes in microbial water quality and health patterns are impacted by the development of these sanitation structures. To substantiate this hypothesis, we retrieved long-term (1990–2017), *in-situ* measurements of annual development of household sanitation units (SAN) for the aforesaid study region (n = 7010 BLKs). SAN has improved from ~60.3 million units in 1990 to ~104 million in 2017, with a linear trend of SAN anomaly (SAN_an_) of 2.63 ± 0.06%/year (1990–2013: 4.09%/year; 2014–2017: 15.15%/year).

**Figure 3:**
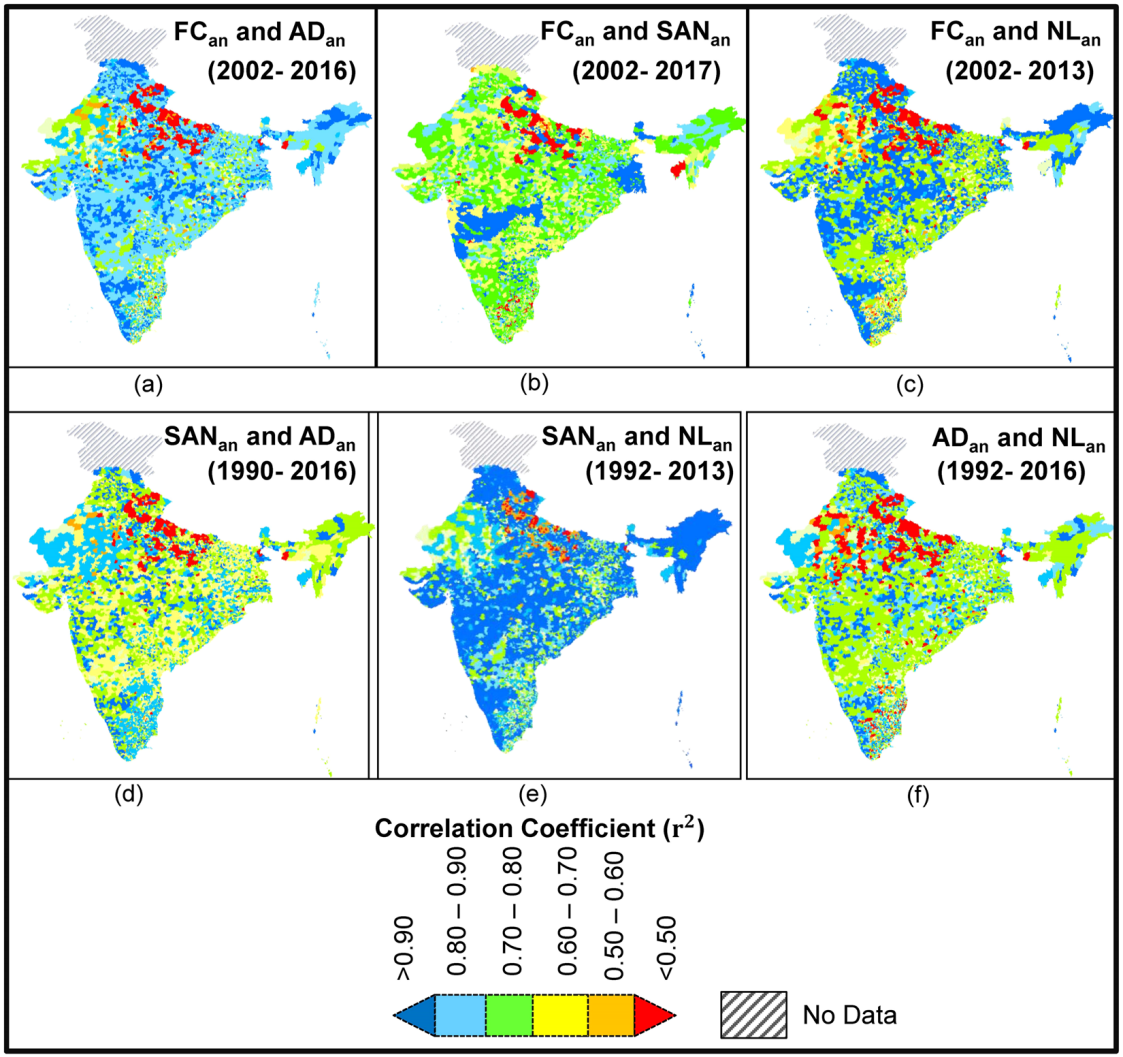
Maps of study area showing correlation for synchronous study periods between different parameters. (**a**) FC_an_ and AD_an_ (2002–2016; r^2^ = 0.985 for entire area, A: 0.99, B: 0.91, C: 0.72 and D: 0.522, p < 0.01), (**b**) FC_an_ and SAN_an_ (2002–2017; r^2^ = 0.922 for entire, A: 0.96, B: 0.84, C: 0.67 and D: 0.77, p < 0.01), (**c**) FC_an_ and NL_an_ (2002–2013; r^2^ = 0.841 for entire, A: 0.96, B: 0.84, C: 0.67 and D: 0.77, p < 0.01), (**d**) SAN_an_ and AD_an_ (1990–2016; r^2^ = 0.895 for entire, A: 0.96, B: 0.84, C: 0.67 and D: 0.77, p < 0.01), (**e**) SAN_an_ and NL_an_ (1992–2013; r^2^ = 0.943 for entire, A: 0.96, B: 0.84, C: 0.67 and D: 0.77, p < 0.01) and (**f**) AD_an_ and NL_an_ (1992–2013; r^2^ = 0.425 for entire, A: 0.96, B: 0.84, C: 0.67 and D: 0.77, p < 0.01)].

Non-linear Hodrick-Prescott trend also indicates an overall increase. SAN_an_ shows significant, strong negative correlation with both FC_an_ (r = −0.96, p < 0.01) and AD_an_ (r = 0.95 p < 0.01) for the simultaneous time periods (i.e., 2002–2017 for FC and 1990–2017 for AD, respectively), thereby supporting our observations that the pathogenic water quality and health condition in the country is improving as a consequence of general improvement of basic sanitation across the country. Thus similar to FC_an_ and AD_an_ trends, discernable spatial variability is identified on the temporal SAN_an_ development patterns. While, >3000 BLKs (44% of study region) show >90% increase in SAN_an_ over the study period, there are still about 1700 BLKs (24%) that show less than 50% development. Significant negative correlations with FC_an_ and AD_an_ (p < 0.01 for both) are also visible in the detailed study areas, with Area A showing a SAN_an_ increase of 96% (SAN_an_ with FC_an_, r = −0.98; SAN_an_ with AD_an_, r = −0.98), followed by B: 81% (r = −0.91, −0.90), C: 62% (r = −0.81, −0.87), and D: 21% (r = −0.74, −0.73), thus demonstrating that sanitation development has not been uniform across the study region (Fig. 3) and a more pervasive plan needs to be undertaken. Lead Lag causality test indicates SAN increase significantly causes decrease in FC and AD in areas A, B, C and D, contemporaneously and in successive years, thereby supporting our hypothesis (Fig. 2b,c, See S2).

To understand the role of land-use change in terms of urbanization; and it’s potential influence on the water quality and public health, at local-to-regional scale, we used annual (1992–2013), satellite based measurement of nightlight (NL)^23^ over the study region. Nightlight data, a well-known and widely used secular proxy of urbanization and sub-national economic development across the globe^24,25^, have been earlier used for identifying water resource allocations^26^. The purpose of it’s application was also to see the applicability of an open-source data like NL, as a rapid proxy for detecting the microbial water quality and health alleviation^27^.

We observed that NL has increased by 89.6% between 1992 and 2013 over the study region, with NL anomaly (NL_an_) linear trend of 4.26 ± 0.05%/year. HP trend also generally increases across the study area, however spatial variability is distinct. While ~63% of the study region (~4500 BLKs, 64.4% of study region) showed >90% increase, potentially indicating economic development, ~15% of the study region (~1100 BLKs, 16% of study region) showed <50% NL increase, thus suggesting less increase in NL as well as economic development. Delineation of trends in the detailed study areas, A, B, C and D, suggest 92.2%, 81.0%, 62.3% and 19.4% improvements of NL, respectively. NL_an_ shows a significant, strong, positive correlation (p < 0.01) with SAN_an_ for entire study region (r = 0.96,) and detailed study areas (A: r = 0.99, B: 0.92, C: 0.91, D: 0.90), indicating areas with ongoing economic development have strong influence on sanitation development. Consequently, NL_an_ show significant negative correlation with FC_an_ (r for entire region −0.92, and r for area A = −0.91, B = −0.85, C = −0.84 and D = −0.84) for 2002–2016, but a relatively weaker correlation with AD_an_ (r for entire area −0.65, r = −0.78, −0.77, −0.72 and −0.64 for areas A, B, C and D). LLC causality test indicate NL increase may significantly lead to SAN increase and can be a strong predictor for decrease in FC and AD in the detailed study area A at contemporary times and successive years, but don’t cause AD in area D (Fig. 2d,e).

We used fixed effect panel data model^27^ to understand the influence of NL and SAN on FC (Model 1; 2002–2017) and AD (Model 2; 1992–2016) for the study region (i.e. across all 7010 BLKs) (Fig. 2f). Absence of endogeneity has been checked by the Hausman specification test^28^. Model 1 demonstrates strong significant impact of both NL and SAN development on water quality (*the coefficient associated with NL*, ${\hat{\beta }}_{NL}$ = −*0*.*004 with t* – *statistics* = −*3*.*41*, [*p* < *0*.*01*] *and SAN*, ${\hat{\beta }}_{SAN}$ = −*0*.*035*, *t-statistic* = −*4*.*365* [*p* < *0*.*01*]), suggesting FC improves with increase in NL and SAN. Results of Model 2 suggest that AD improves with SAN (${\hat{\beta }}_{SAN}$ = −*0*.*63*, *t-statistic* = −*2*.*69* [*p* < *0*.*01*]) but not necessarily with NL (${\hat{\beta }}_{NL}$ = *0*.*06*, *t-statistic* = *0*.*78* [*p* < *0*.*01*]). To calculate the causal impact of NL and SAN on AD and FC for highly improved areas (i.e. detailed study area A), and less improved areas (i.e. detailed study area D) we used LLC test, which suggest that while in areas with highly improved FC (e.g. detailed study area A), NL and SAN are significant predictor for decrease in AD. However, in areas with less improved FC (e.g. detailed study area D) NL was not found to be a strong predictor for AD (See S2).

These disparities suggest that urbanization (represented by NL) and sanitation development (SAN), together described as Development (DEV) may not solely result to alleviation of water quality (FC) and water-borne diseases (AD), together described as Water Quality and Health (WQH) in across the study region over India. Thus, we infer that other factors (e.g. improper human practice) may have stronger influence in some localities (e.g. detailed study areas C and D). It has been reported that many of the millions of basic sanitary structures built across India during several decades, exist in dilapidated condition^3,8^. Also, several communities across South Asia, historically perceive house-hold sanitation structures as impure and unhealthy solutions to more preferred, (open) defecation away from home.

**Figure 4:**
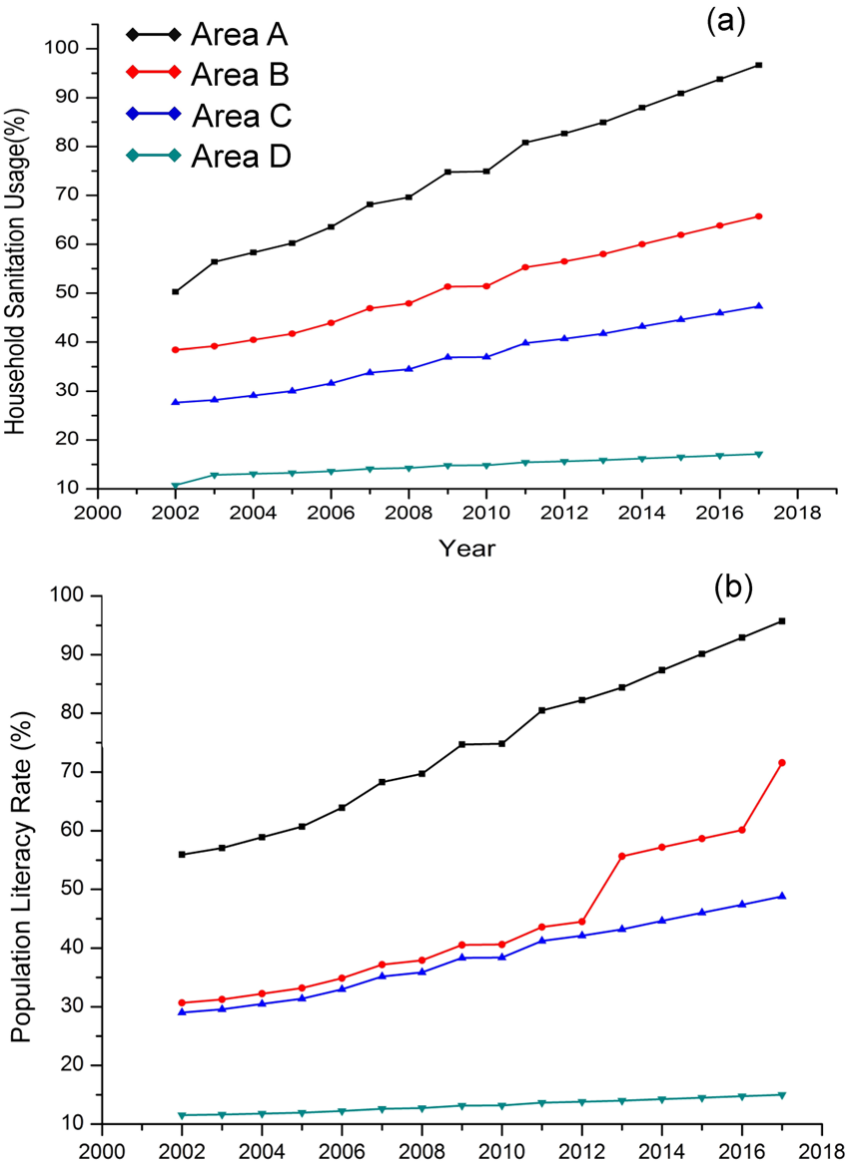
Temporal trends of (**a**) Household Sanitation usage (SANu) and (**b**) Literacy rate (Lt) as percentage of population in the detailed study areas A through D.

**Figure 5:**
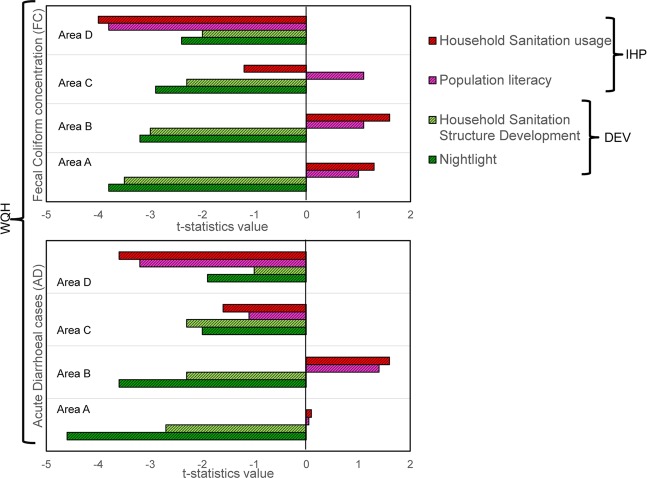
Bayesian VAR t-statistics value, showing impact of Sanitation and Economic development (DEV) and Improper Human Practices (IHP) on Water Quality and Health (WQH) for detailed study area A through D. Positive and negative t-statistics value indicates more direct and inverse, significant variance relationships, respectively.

Thus, to quantify these factors, we retrieved temporal data on SAN usage (*SAN*_*u*_) and population literacy (*Lt*) data for the detailed areas A, B, C and D (Fig. 4), for delineating the influence of improper human practices (*IHP*) of water quality and health. SAN_u_ data includes population, who are not using sanitation structures in spite of their presence (see SI), for all relevant causes^29^. Lt includes population with primary education^30^ (see SI). We used IHP parameters (SAN_u_ and Lt) and DEV (SAN and NL) in first-order Bayesian Vector Auto regression (BVAR) analyses for the detailed study areas A,B,C and D (Fig. 2g) to elucidate potential impact on WQH (FC and AD). In areas, where NL doesn’t seem to be a strong predictor for AD (i.e. detailed study area D), IHP is found to have significant (p < 0.01) negative impact on WQH. This suggest that in the areas with less development, improper human practices are predominating factor in causing decline in groundwater quality and increasing enteric disease burden. In contrast, in detailed study area A, where NL and SAN strongly influence in reduction of AD, BVAR analyses suggest minimal influence of IHP on AD. Detailed areas B and C demonstrate interim results of those between detailed study areas A and D (Fig. 5). Thus, we are able to delineate and quantify the probable causes for the evolving trends of water-borne pathogens and related enteric disease in the detailed study areas A, B, C and D.

**Figure 6:**
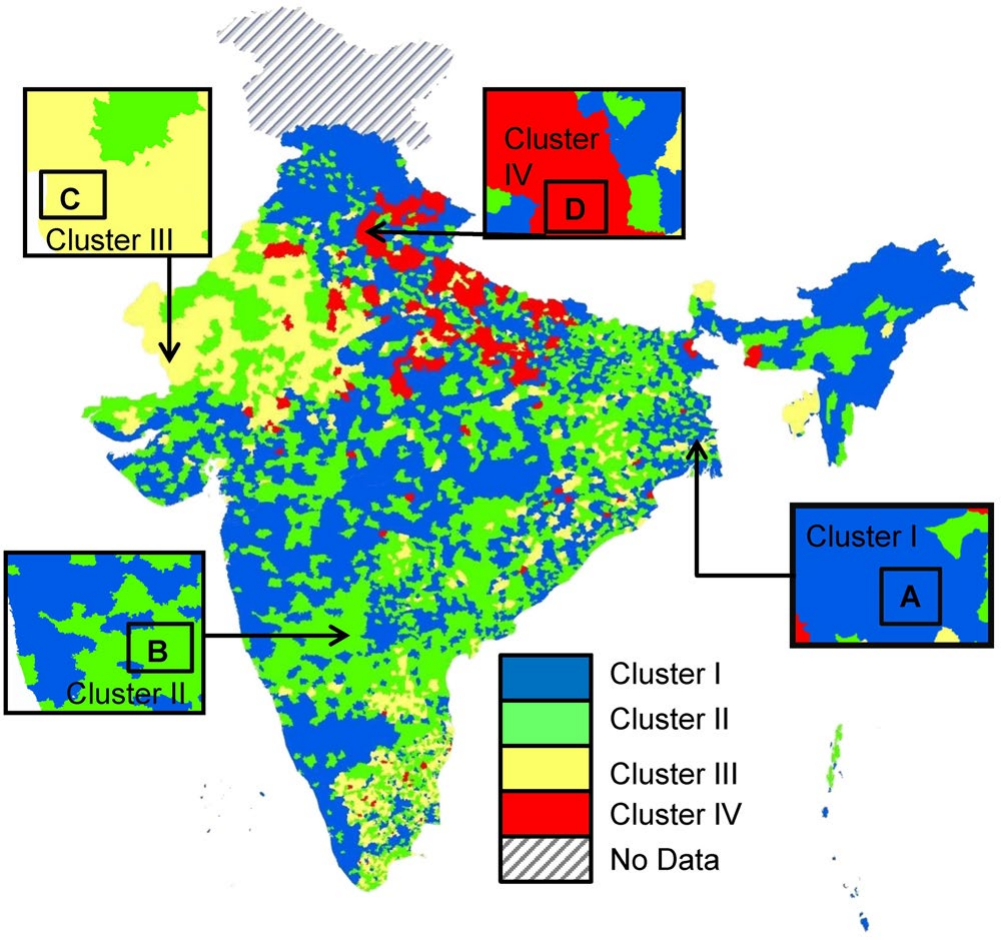
Map of the study area showing the four clusters, showing *Cluster I* (superset of detailed study area A) blocks (3069 BLKs, i.e. 44% of entire study region) where Water Quality and Health (WQH) alleviation are predominantly influenced by Sanitation and Economic development (DEV), and Improper Human Practices (IHP) has minimal influence, within the study period; *Cluster II* (superset of area B) blocks (1596 BLKs, 23%), where DEV influence WQH, but IHP has some effect; *Cluster III* (superset of area C) blocks (631 BLKs, 9%), where IHP influence WQH, but DEV has some effect; and *Cluster IV* (superset of area D) blocks (1714 BLKs, 24%), where IHP predominant influence on WQH, but DEV has minimal influence.

In order to up-scale our observations of relation of IHP and DEV on WQH for the entire study region (i.e. all 7010 BLKs), we applied multivariate cluster analyses (Fig. 2h) for the whole study region (n = 7010 BLKs) by including the FC, AD, SAN and NL data (See S2). Our analyses delineate four major clusters, where *Cluster I* becomes a superset of area A (3069 BLKs, i.e. 44% of entire study region), *Cluster II* of area B (1596 BLKs, 23%), *Cluster III* of C (631 BLKs, 9%) and *Cluster IV* for D (1714 BLKs, 24%). Figure 6 demonstrates the spatial locations of the Cluster I, II, III and IV BLKs, such that Cluster I are areas where DEV reduces WQH, and IHP has minimal influence. On the contrary, locations of Cluster IV suggest the areas where IHP has influenced in decline of WQH, and DEV has minimal influence.

Thus, our results show that each of the 7010 BLKs has very unique condition, and the policy makers would have to prepare customized strategies for providing access to clean drinking water to all of the residents within the study region. Also, just a pervasive economic development and/or sanitation development may not be sufficient for overall societal development across the study areas. Other, less visible and indirect influencing parameters can have very strong influence on health and water quality decline, and those needs to be quantified and addressed properly. For example, several of the Cluster IV areas include suburbia and peri-urban areas, where rapid urban expansion is happening. These areas typically have very little or no previous SAN, however, a sudden migration of huge population can lead to unsustainable human living conditions. From this study, such situation are visible in eastern (mostly western Uttar Pradesh state) and western (mostly eastern Uttar Pradesh state) of the capital city of New Delhi.

In a diverse country like India, where there is tremendous heterogeneity between geology, climate, landuse, human groundwater usage, economic prosperity, religious and social practices, it is extremely difficult to prescribe a policy that can be applicable to all. It is well known that declining water quality has impacted health of a major part of the Indian population. But there are no identified mechanisms to find out such non-pollution sources, as well as identifying the social and economic developments that can be predictor for such pollution. The outcome of this study may provide the first integration and analyses of the dataset and provide geo-spatial indicators that can help to identify and target the areas, where development is insufficient to alleviation of water quality vis-à-vis water borne diseases and can help to plan strategies accordingly. More importantly, this is one of the first studies, where the importance of less visible factors likes urbanization and literacy rates are included in quantification for decline in water quality and health. Hence, it is imperative that the policy makers need to take more intricate look at the economic aspects, as well as human factors and societal fabric to evaluate the success of water quality improvement plans. For example, in some areas of Cluster III and IV, nudging for improving human behavior (e.g. sanitation usage) may be a stronger mechanism of water quality alleviation than economic development. Similarly, there are other areas (mostly Cluster IV), where the nature of the population (e.g. transitory versus settled) can result to substantial impact on water-borne diseases. Further, the observation of our study is valid at the scale of our observation i.e. block scale, and finer observation granularity or scale can lead to identification of other inherent factors. For example, the influence of hydrological factors (specifically subsurface hydrogeology) was not investigated in the present study. However, we understand that in a finer-scale, the degree of lateral and vertical separation of waste can have substantial impact on water quality, independent of either DEV or IHP, and thus needs to be studied in details. Hence, for providing access to safe water and sanitation, which are the core of sustainable development and survival of the residents across the study region^30,31^, detailed integration between scientific understanding, economic improvement and societal development is required^32,33^.

## Conclusion

The United Nation Millennium Plan for Sustainable Development has identified access to safe water and basic sanitation, along with good health and wellbeing for all by 2030, as their primary Goals 4 and 6^1^. The plan also includes poverty alleviation and its consequent effects as Goal 2^1^. At present, about a third of the world population (>2 billion, mostly in poor countries) is still waiting for achieving these goals, of which >500 million of these people live in India who still practice open-defecation^3^. Over the last few decades and specifically in last few years, administrative authorities have implemented policies of developing millions of sanitation structures but their efficacy on improving water quality or consequent health condition have not been understood. In this article, we use spatio-temporal patterns, multivariate statistical models and causality tests to show that more than decade long, annual decrease in *in-situ* measured groundwater fecal pathogen concentrations (**−**3.09%/year, 2002–2016) and about three decades of acute diarrhoea cases (−2.69%/year, 1990–2017) in the spatial resolution of smallest administrative land units (blocks or equivalents, n = 7010) across major parts of Indian region. However, it is yet to achieve the goal of no-FC clean drinking water. They have been significantly caused and impacted by house-hold sanitation development (9.62%/year, 1990–2017), in contemporary and successive years. Since 2014, it has been observed that the FC concentrations have reduced at an enhanced rate of 2.33%/year and AD has alleviated at the rate of 2.96%/year. The sanitation coverage has also increased at the rate of 22.5%/year since 2014. We also demonstrate that such sanitation and water quality improvement (3069 BLKs)are caused and impacted by urbanization and land-use change as suggested by increasing satellite-based night-time light (9.15%/year, 1992–2017). We also observe that such secular data, like NL can be effectively used, in most areas (>80%) as a predictor for water quality changes and alleviation of health case, in places where intensive, high resolution *in-situ* data of water quality and health are unavailable. However, testing the applicability of such proxy in other places can increase its acceptability for wider usage. We conclude that in the last three decades, groundwater fecal pathogen and associated acute diarrhoea cases generally improved in most areas of India, and has been mostly caused by sanitation development, urbanization and related-land use changes. However, external factors like societal practices linked to education level, proper human practices, etc., can also exert major influence on water-borne diseases loads of an area Enhanced alleviation of water quality and health due to drastic decrease of groundwater faecal coliform concentration were observed since inception of Clean India (Swachh Bharat) Mission in 2014, which lead to improved construction of sanitation constructions across the country. However, studies for more extended time period is required for providing conclusive insights. For better results of policy interventions on groundwater-based drinking water quantity and quality, integration of scientifically-prudent economic and societal development is required.

## Methods

### Data acquisition and management

#### Water quality, sanitation, human practices and health

In order to assess the long-term trends in changing microbial groundwater quality, health and sanitation coverage over major parts of India, we retrieved total measurements (n = 1,726,233) of annual concentrations of Faecal Coliform concentrations in groundwater (FC) from 7010 administrative blocks or its equivalent (BLK) across the study domain (Fig. 1). A block is a district sub-division, defined for the purpose of government land administrative purpose, and is considered as the smallest unit of the Indian administration division. The data was screened for temporal continuity and 5,88,840 continuous measurements were considered for final statistical analyses for the study period (2002–2017). These measurements were scaled up to the aforesaid geo-tagged 7010 BLKs. For each year, each block had a median of 42 measurements (minimum: 5; maximum: 79). All of the measurements for each block have been up scaled to block level by taking their median value. To study this FC spatial variability, its cause/s and consequent human health impacts, in details, we selected high-resolution study area clusters of 30 BLKs within the study region (see SI). While, FC concentrations were retrieved as point measurements in sub-block scale, measurements for Acute Diaharea (AD) cases and house-hole sanitation structures (SAN) were available in block-scale. Continuous data for AD (1990–2016) and SAN (1990–2017) in block scale have been considered for the study period.

We retrieved long-term (annual, AD and for 7010 BLKs (Fig. 1) for the study area. The retrieved data were cleaned and culled for continuity and robustness. In order to make the data statistically robust, third quartile was calculated for each block for all the years, for a particular parameter^31^. In order to omit outliers in the data, Tukey’s method (1977)^34^ was used, where the outlier limit was calculated by multiplying the third quartile value with 1.51. The data exceeding the outlier limit were ignored whereas the values lesser than this limit were kept unaltered. The resulting set of cleaned data for the study domain and period was used for the study and various analyses, and the results were plotted in a map using the ARC GIS (10.2). Sanitation usage and Literacy rate data were also used for areas A through D.

#### Night-time light

The Operational Linescan System (OLS) sensors located at the satellites from US Air Force Defense Meteorological Satellite Program (DMSP) are designed to observe clouds, cloud top temperatures, nighttime satellite coverage at global scale. The National Geophysical Data Center (NGDC), a subsidiary of the National Oceanic and Atmospheric Administration (NOAA), have processed the lighting associated with human activities at a global-scale and released the data with less than 1 km (30 arc-second) spatial resolution across the globe at an annual scale between 1992 and 2013 (Table S1). They have processed the data for removing the signals from clouds, moonlight, seasonally late sunsets and auroral events. Filtered observations over the days in a year are averaged and converted to “digital value” between 0 and 63, where 0 represents no lighting condition and 63 represents maximum possible lighting condition. The uniqueness of the data allows user to link the nightlight related information with economic activity at a high spatial resolution (sub-State level or more). As a result, several past studies have investigated the link between nightlight and economic activities. We have acquired the data for the Indian region at the highest possible resolution (30 arc-second) from the NGDC archive between 1992 and 2013. In order to compare with other parameters (i.e. water quality, health related parameters etc. that are available at block-scale), the data are processed at administrative block level (number of blocks used = 7010). Nightlight digital values from all of the pixels within each administrative block are spatially averaged and the block level nightlight data has been generated across India (Fig. SI).

### Statistical analysis

#### Panel data analyses

Panel data analyses were conducted to estimate heterogeneity for given measure of SAN and NL against AD and FC. The comparison between the dependent and the independent variable is done by means of t- statistics. SAN and NL were considered as independent variables and AD and FC were counted as dependent variables in fixed effect panel data analyses. To quantify the relationship and identify the impact of NL and SAN separately on FC and incidence of AD, we have estimated fixed effect model. Details of the panel data analysis has been described in S2.1. Panel data analyses were conducted using STATA v. 13 statistical software.

Model 1: To quantify the impact of NL and SAN on FC, we have regressed FC on NL and SAN using 7010 blocks for the time period of 2002–2013. Result of fixed effect shows that NL and SAN have significant negative impact on FC (Table S2a). This implies that water quality improves with both development and household sanitation structures. The fixed effect model reported in Table S1a has good fit with r^2^ = 0.93. Presence of endogeneity has been checked by the Hausman specification test^7^. Here the Hausman specification test statistic follows chi-square distribution with 2 degrees of freedom. The calculated value of the Hausman specification test is 47012 tabulated values of 5.991 at 5% significance level. Therefore, the Hausman specification test rejects the null hypothesis and shows that there is no endogeneity. As a result, we will concentrate on fixed effect model as it gives consistent estimates in absence of endogeneity.

Model 2: To quantify the impact of nightlight and sanitation coverage on acute diarrheal cases, we have regressed acute diarrheal cases on nightlight and sanitation coverage using 7010 blocks for the time period of 1992–2013. Result of fixed effect shows that sanitation coverage has significant negative impact on acute diarrheal cases but nightlight does not show significant impact on it. This implies that acute diarrheal cases improve with household sanitation structure unlike nightlight (Table S2b,c). The fixed effect model reported in table S2b has moderate fit with r^2^ = 0.89. Presence of endogeneity has been checked by the Hausman specification test. Here the Hausman specification test statistic follows chi-square distribution with 2 degrees of freedom. The calculated value of the Hausman specification test is 10124 > tabulated value of 5.991 at 5% significance level. Therefore, the Hausman specification test rejects the null hypothesis and shows that there is no endogeneity. It suggests fixed effect model gives consistent and efficient estimate in absence of endogeneity.

#### Multivariate cluster analyses

Multivariate statistics (hierarchical cluster analyses, HCA) was done on the original data set (without any weighting or standardization). HCA was performed on the FC, AD, SAN and NL for each location (n = 7010 Blocks). The HCA dendogram was constructed by Ward’s method with squared Euclidean distance. HCA was used to investigate relationships between the locations. Below detection level (bdl) measurements were replaced by dl × 0.55 for calculation^26^. HCA was analyzed by E-views v 9.5 statistical software. Four clusters were identified. Details of the outcome of the analysis are elaborated in S2.3 (Fig. S4).

#### Hodrick prescott filtering and trend analyses

The Hodrick-Prescott (HP) filter, a non-parametric trend estimator, has been used in this study for computing the trend analysis^13^. The trends and cycles are separated in this approach upon solving the following equation, where T_t+1_ and T_t−1_ are the trend component at the time steps *t* + *1* and *t* − *1*, respectively. The cyclical components can be obtained after removing the trends from its real values; also, the long-term mean of the cyclical component closes to zero. Variability in cyclical components is reduced through the selection of a suitable value for the smoothing parameter (λ). The selected value of λ for annual data is 600.

1 $${\rm{Min}}\,({\rm{T}})\,\mathop{\sum }\limits_{{\rm{t}}=1}^{{\rm{T}}}\,({({{\rm{y}}}_{{\rm{t}}}-{{\rm{T}}}_{{\rm{t}}})}^{2}+{(({{\rm{T}}}_{{\rm{t}}+1}-{{\rm{T}}}_{{\rm{t}}})-({{\rm{T}}}_{{\rm{t}}}-{{\rm{T}}}_{{\rm{t}}-1}))}^{2}$$

#### Bayesian VAR analyses

We have estimated Bayesian VAR for area A, B, C and D. Lag values of household both sanitation development (SAN) and night-time light (NL) have a significant (p value < 0.01) negative impact on fecal coliform concentration (FC) and acute diarrheal cases (AD) in area A, B, C but not in area D. Improper human practices (IHP) which includes sanitation usage and accessibility and literacy levels have a significant (p value < 0.01) positive impact on FC and AD in area D (Table S3). The accumulated impulse of BVAR is given in Fig. S3.

#### Lead - lag correlation test

We study the lead/lag relationships by computing three lagging indicators using E-views v. 9.5 statistical software. We have analysed LLC of NL and SAN on AD and IHP. We have analysed LLC of NL and SAN on AD and IHP on AD by choosing proper lag length using the following equation:

2 $${{\rm{p}}}_{{\rm{\max }}={[12\ast \frac{T}{100}]}^{1/4}}$$

Where, T is the total number of usable observations after adjusting for lags. We need to keep the total number of usable observations unchanged under different lag length for selecting optimal lag length. We continue our analysis by decreasing the lag length by one but keeping the number of observations unchanged. We have chosen the optimal lag length 3 as it gives us least SBIC (Schwarz Bayesian Information Criterion). Our model selection is based on SBIC as it always chooses most parsimonious model.

### Assumptions and uncertainty

All of the data used here are not available for a continuous study period, leading to use of maximum possible data range as per the availability from respective agencies. The comparisons between the dataset are done for mutually available time-period (Table S1). The data representativeness is dependent on the data provided by the government sources, we have applied possible statistical tests for filtering the data in order to upscale it from individual data location to block-scale and analyses further using the block-scale data.

*Some other assumptions and limitations are provided below*:

1. Strict exogeneity: E[∈it|Xi, αi] = 0E[∈it|Xi, αi] = 0 (In words, the idiosyncratic errors are uncorrelated with the covariates and the fixed effects).
2. No multi-collinearity: This is why we can’t have time constant covariates in X; they would be collinear with the fixed effect which is also time invariant.
3. Idiosyncratic error is uncorrelated and homoscedastic [This is not really true because by de-trending the errors, we have introduced some dependence, to get around this we need to use the Huber White sandwich estimator (another topic of its own) to adjust the standard errors for ββ].

## Supplementary information

Supplementary Information

## Supplementary information

is available for paper at 10.1038/s41598-019-50875-w.
