# Author Correction: Impact of sanitation and socio-economy on groundwater fecal pollution and human health towards achieving sustainable development goals across India from ground-observations and satellite-derived nightlight

**DOI:** 10.1038/s41598-019-55236-1

**Published:** 2019-12-04

**Authors:** Abhijit Mukherjee, Srimanti Duttagupta, Siddhartha Chattopadhyay, Soumendra Nath Bhanja, Animesh Bhattacharya, Swagata Chakraborty, Soumyajit Sarkar, Tilottama Ghosh, Jayanta Bhattacharya, Sohini Sahu

**Affiliations:** 1 School of Environmental Science and Engineering, Indian Institute of Technology, Kharagpur, India; 2 Department of Geology and Geophysics, Indian Institute of Technology, Kharagpur, India; 3 Department of Humanities and Social Sciences, Indian Institute of Technology, Kharagpur, India; 4 Faculty of Science and Technology, Athabasca University, 1 University Dr, Athabasca, AB T9S 3A3 Canada; 5 Water and Sanitation Support Organization, Public Health Engineering Department, Govt. of West Bengal, Kolkata, India; 6 Cooperative Institute for Research in Environmental Sciences (CIRES), CU, Boulder; Earth Observation Group, NOAA National Centers for Environmental Information, Boulder, Colorado United States; 7 Department of Mining Engineering, Indian Institute of Technology, Kharagpur, India; 8 Department of Economic Sciences, Indian Institute of Technology, Kanpur, India

Correction to: *Scientific Reports* 10.1038/s41598-019-50875-w, published online 23 October 2019

In the original version of this Article, Tilottama Ghosh was incorrectly affiliated with ‘Earth Observation Group, National Geophysical Data Center, National Oceanic and Atmospheric Administration, Maryland, USA’. The correct affiliation is listed below.

Cooperative Institute for Research in Environmental Sciences (CIRES), CU, Boulder;

Earth Observation Group, NOAA National Centers for Environmental Information, Boulder, Colorado.

This error has now been corrected in the PDF and HTML versions of the Article, and in the accompanying supplementary material.

