# Supplementary material for: Impact of sanitation and socio-economy on groundwater fecal pollution and human health towards achieving sustainable development goals across India from ground-observations and satellite-derived nightlight
Source: Sci Rep. 2019 Oct 23;9:15193. doi: 10.1038/s41598-019-50875-w (PMC6811533; doi:10.1038/s41598-019-50875-w)
Supplement: Supplementary file 1 — Supplementary Information [file 41598_2019_50875_MOESM1_ESM.docx]

**Supplementary Information**

**Impact of sanitation and socio-economy on groundwater fecal pathogen pollution and human health across India from ground-based observations and satellite-derived nightlight**

Abhijit Mukherjee^1,2,*^, Srimanti Duttagupta^1^, Siddhartha Chattopadhyay^3^, Soumendra Nath Bhanja^4^, Animesh Bhattacharya^1,5^, Swagata Chakraborty^2^, Soumyajit Sarkar^1^, Tilottama Ghosh^6^, Jayanta Bhattacharya^1,7^, Sohini Sahu^8^

1School of Environmental Science and Engineering, Indian Institute of Technology, Kharagpur, India 2Department of Geology and Geophysics, Indian Institute of Technology, Kharagpur, India 3Department of Humanities and Social Sciences, Indian Institute of Technology, Kharagpur, India

4Faculty of Science and Technology, Athabasca University, 1 University Dr, Athabasca, AB T9S 3A3, Canada

5Water and Sanitation Support Organization, Public Health Engineering Department, Govt. of West Bengal, India

6 Cooperative Institute for Research in Environmental Sciences (CIRES), CU, Boulder;

Earth Observation Group, NOAA National Centers for Environmental Information, Boulder, Colorado.

7Department of Mining Engineering, Indian Institute of Technology, Kharagpur, India

8Department of Economic Sciences, Indian Institute of Technology, Kanpur, India

*****Correspondence to [amukh2@gmail.com,](mailto:amukh2@gmail.com) [abhijit@gg.iitkgp.ac.in](mailto:abhijit@gg.iitkgp.ac.in)

# Contents of Supplementary Information

**S1 Household sanitation development, acute diarrhoeal cases and fecal coliform concentration and nightlight data acquisition**

**S2Statistical analyses**

**S1 Household sanitation development, acute diarrhoeal cases and fecal coliform concentration and nightlight data acquisition**

We have used datasets as per the availability of each parameter collected from respective Ministries (Table S1). The data values exceeding the outlier limit were ignored whereas the values lesser than this limit were kept unaltered. In order to assess the potential changes of water quality long term fecal coliform concentration data (2002 – 2017) from major parts of India (n_max_

= 7010) were studied in details. Anomalies for individual years have been spatially plotted for household sanitation development anomalies (SAN_an_), acute diarrheal cases anomalies (AD_an_) and fecal coliform concentration anomalies (FC_an_) [Fig S1a, b and c].

Selected high-resolution study area clusters of 30 BLKs within the study region, corresponding to each of *highly improved* (Area A, latitude: 88.297°E to 88.764°E, longitude: 22.357°N to 22.864°N) *improved* (Area B, latitude: 75.505°E to 76.59°E, longitude: 20.822°N to 22.05°N), *moderately improved* (Area C, latitude: 71.489°E to 73.154°E, longitude: 25.458°N to 27.046°N) and *less improved* (Area D, latitude: 77.637°E to 78.939°E, longitude: 29.249°N to 30.596°N) areas.

**Table S1:** Entire time period, spatial coverage and source of data collection for FC, SAN, AD and NL

| Data | Spatial Coverage | Temporal coverage | Source |
| --- | --- | --- | --- |
| Water quality (FC) | 7010 Blocks from  ~170k habitations | 2002 - 2017 | Public Health Engineering Department, Ministry of Drinking water and sanitation |
| Household Sanitation Structure (SAN) | 7010 Blocks | 1990 - 2017 | Public Health Engineering Department, Ministry of Drinking water and sanitation |
| Acute Diarrheal Cases (AD) | 7010 Blocks | 1990 – 2016 | Ministry of Health and family welfare |
| Night-time light (NL) | 7010 Blocks from ~900 m pixels | 1992 – 2013 | NASA |

**Fig. S1:** (a) Spatial distribution of household sanitation development anomalies (SAN_an_) between 1990 and 2017. Maps were made using ArcGIS 10.2.


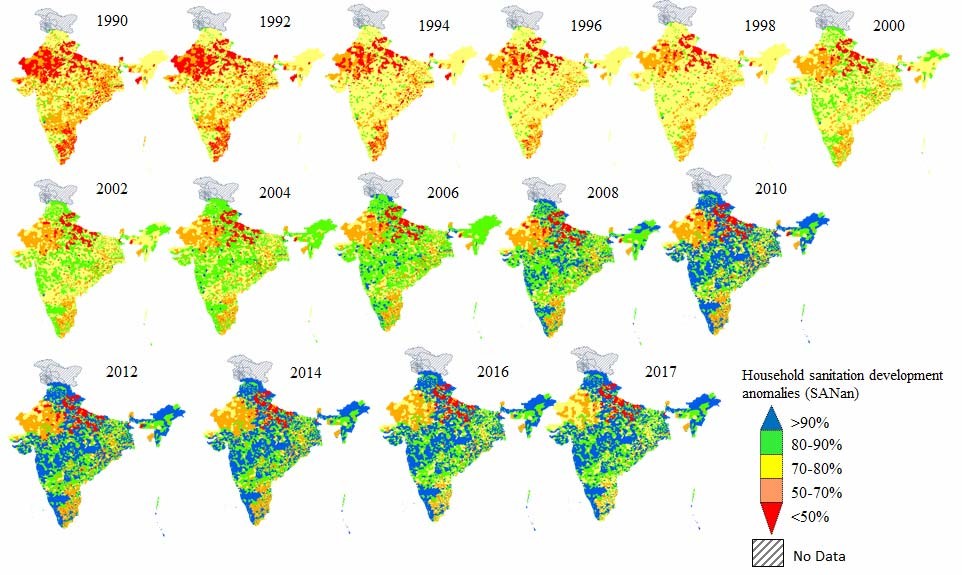


**Figure S1:** (b) Spatial distribution of acute diarrheal cases anomalies (AD_an_) between 1990 and 2016. Maps were made using ArcGIS 10.2.


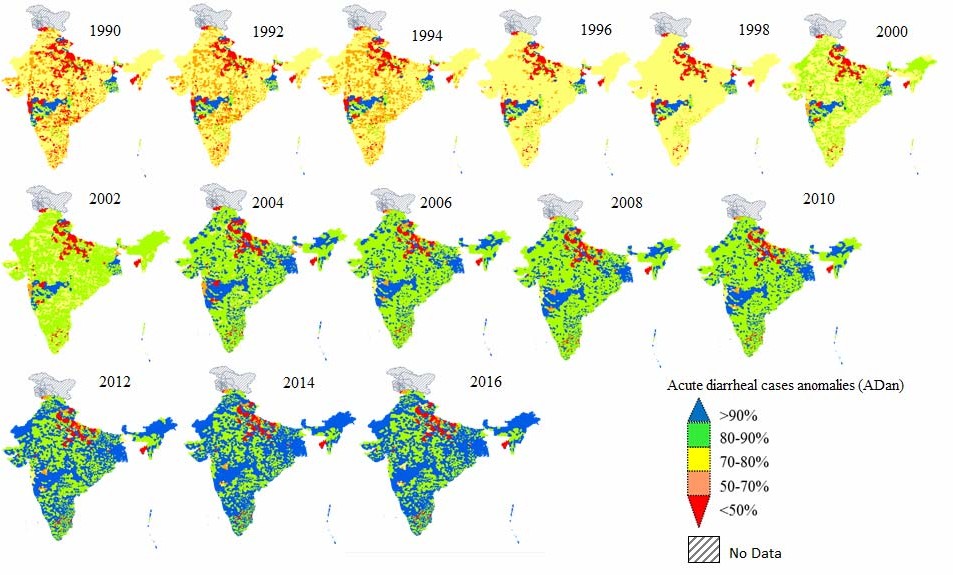

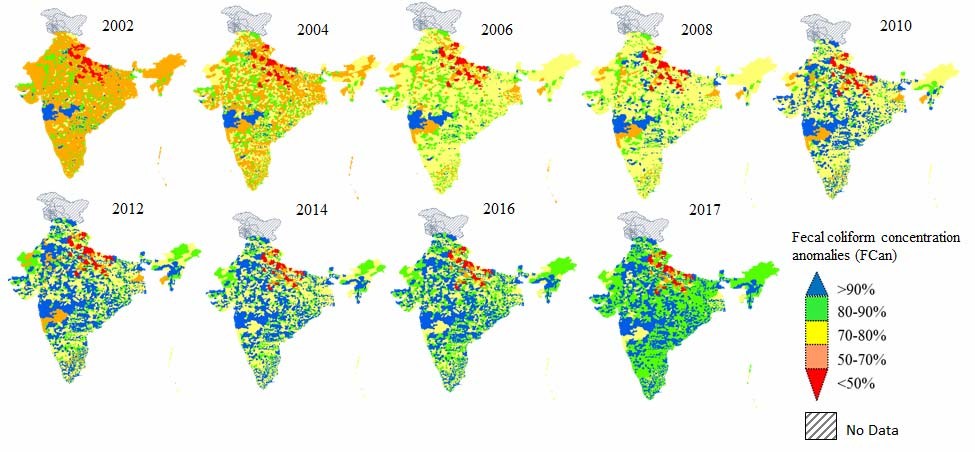


**Figure S1(c):** Spatial distribution of fecal coliform concentration anomalies (FC_an_) between 2002 and 2017. Maps were made using ArcGIS 10.2.

Nightlight digital values from all of the pixels within each administrative block are spatially averaged and the block level nightlight data has been generated across India (Figure S2).


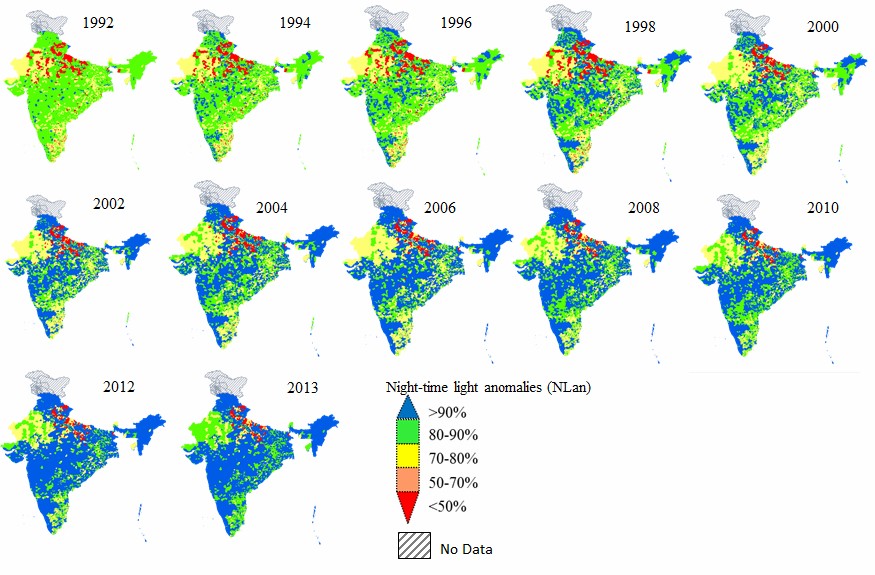


**Figure S2:** Spatial distribution of night-time light anomalies (NL_an_) between 1992 – 2013. Maps were made using ArcGIS 10.2.

# S2 Statistical analyses S2.1 Panel data analyses

NL is a public good which is non-excludable in nature. Therefore, NL creates positive externality in the sense. Here, we used NL as a measure of development. This implies once an area becomes developed, the fruit of development is enjoyed equally by everybody. Therefore, we have used night-time light (NL), household sanitation structure (SAN), fecal coliform concentration (FC) and acute diarrheal cases (AD) for panel data analysis.

**Table S2a:** Panel data analysis showing fixed effect of NL and SAN on FC

| Dependent Variable: FC | |
| --- | --- |
| Independent Variable | Fixed Effect |
| NL | -0.004 |
|  | (-3.41)^*^ |
| SAN | -0.035 |
|  | (-4.365)^*^ |
| Constant | 14.018 |
|  | (15.628)^*^ |
| No. observation | 70110 |
| r2 | 0.927 |
| Hausman Specification Test | |
| Hausman: Chi^2^(2) calculated | 47012.05 |
| Hausman: Chi^2^(2) tabulated (5%) | 5.991 |
| Test for Block specific effect | |
| F (7010, 63099) Calculated | 24501.05 |

*t-statistics values are shown in parenthesis.

**Table S2b:** Panel data analysis showing fixed effect of NL on AD

| Dependent Variable: AD | |
| --- | --- |
|  | Fixed Effect |
| SAN | -0.63 |
|  | (-2.69)^*^ |
| Constant | 29.03 |
|  | (49.36)^*^ |
| No. observation | 70110 |
| r2 | 0.895 |
| Hausman Specification Test | |
| Hausman: Chi^2^(2) calculated | 31205.660 |
| Hausman: Chi^2^(2) tabulated (5%) | 5.991 |
| Test for Block specific effect | |
| F (7010,63099) Calculated | 17596 |

*t-statistics values are shown in parenthesis.

**Table S2c**: Panel data analysis showing fixed effect of NL on AD

| Dependent Variable: AD | |
| --- | --- |
|  | Fixed Effect |
| NL | 0.06 |
|  | (0.78)^*^ |
| Constant | 13.02 |
|  | (19.33)^*^ |
| No. observation | 70110 |
| r2 | 0.236 |
| Hausman Specification Test | |
| Hausman: Chi^2^(2) calculated | 2130.25 |
| Hausman: Chi^2^(2) tabulated (5%) | 5.991 |
| Test for Block specific effect | |
| F (7010,63099) Calculated | 14501 |

*t-statistics values are shown in parenthesis.

Constant measures block specific heterogeneity. The result implies block specific effect is also statistically significant. It may include state specific and block specific government policies, religion, best practice, awareness and education etc which are unobserved. To test the block specific importance we have performed F-test. In each model, F test calculated values (24501, 17596 and 14501 respectively) are way higher than the tabulated value at 5% significance level (1.89).

# S2.2 BVAR and Lead-lag causality analyses

We have estimated Bayesian VAR and tested Lead-lag causality for area A, B, C and D. Lag values of household both sanitation development (SAN) and night-time light (NL) have a significant (p value < 0.01) negative impact on fecal coliform concentration (FC) and acute diarrheal cases (AD) in area A, B, C but not in area D. Improper human practices (IHP) which includes sanitation usage and accessibility and literacy levels have a significant (p value <0.01) positive impact on FC and AD in area D (Table S3).

**Table S3**: Bayesian VAR parameters [t-statistics value] for four areas (area A, B, C and D)

| Area A | | | Area B | | Area C | | Area D | |
| --- | --- | --- | --- | --- | --- | --- | --- | --- |
|  | FC | AD | FC | AD | FC | AD | FC | AD |
| NLt-1 | -0.256  [-3.254]^*^ | -0.1258  [-2.661]^*^ | -0.215  [-2.95]^*^ | -0.1089  [-2.159]^*^ | -0.208  [-2.025]^*^ | -0.0953  [-2.122]^*^ | -0.189  [-1.953] | -0.623  [-1.056] |
| SAN_t-1_ | -0.513  [-3.586]^*^ | -0.186  [-4.623]^*^ | -0.362  [-3.092]^*^ | -0.095  [-3.396]^*^ | -0.265  [-2.623]^*^ | -0.079  [-2.029]^*^ | -0.196  [-2.09]^*^ | -0.061  [-1.933] |
| Ltt-1 | 0.053  [1.09] | 0.023  [0.03] | 0.569  [1.162] | 0.209  [1.226] | 0.322  [1.241] | 0.262  [-1.096]^*^ | 0.904  [-2.95]^*^ | 0.559  [-3.05]^*^ |
| SAN_ut-_  1 | 0.058  [1.32] | 0.022  [0.07] | 0.219  [1.512] | 0.42  [1.339] | 0.61  [-1.08] | 0.72  [-1.69]^*^ | 0.89  [-3.85]^*^ | 0.87  [-3.52]^*^ |

t-statistics values are shown in []. * Statistically significant at 99% confidence level

**Figure S3:** Accumulated Impulse of Bayesian VAR for FC, AD, NL and SAN


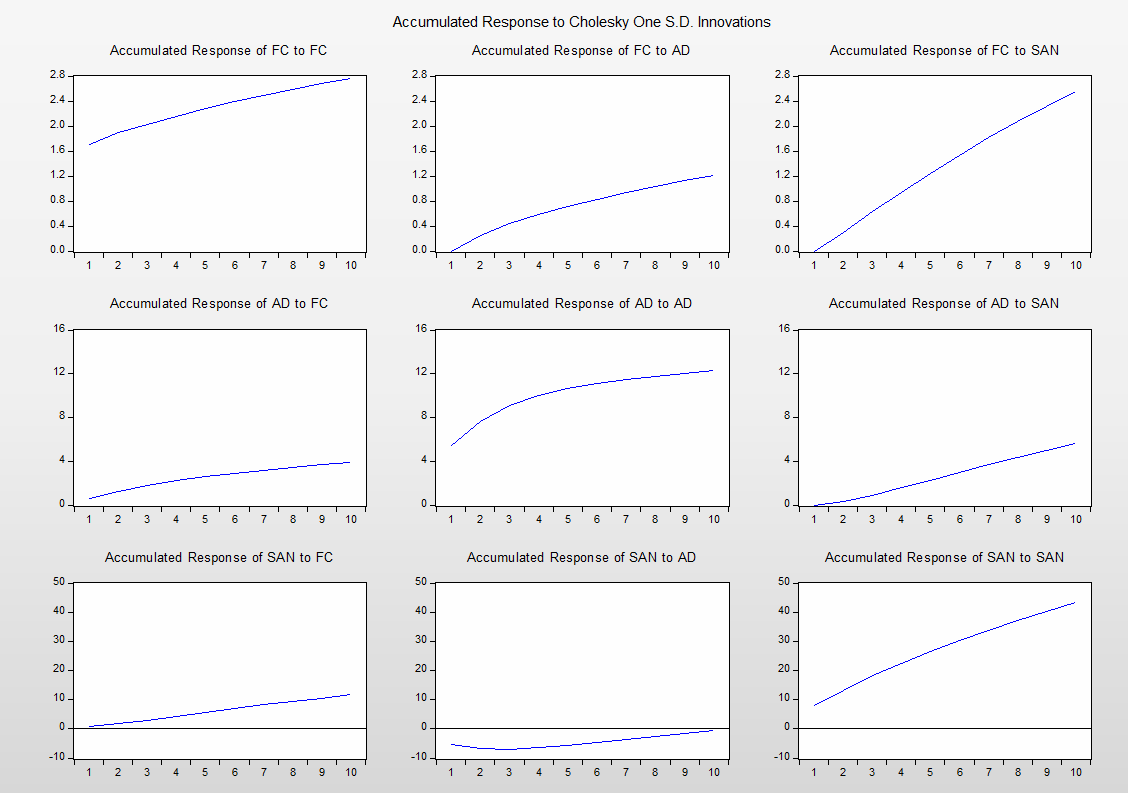


# S2.3 Multivariate Hierarchical Clustering Analyses

According to multivariate cluster analysis study area was divided into four clusters (Cluster I, II, III and IV). Cluster I, II, III and IV are the superset of area A, B, C and D respectively. Cluster I belongs to area which are economic development predominated. Cluster II belongs to economic development dominated area, cluster III belongs to improper human practice dominated areas and cluster IV belongs to the area which is improper human practice predominated.

**Figure S4:** Multivariate hierarchical cluster analysis for the whole study area


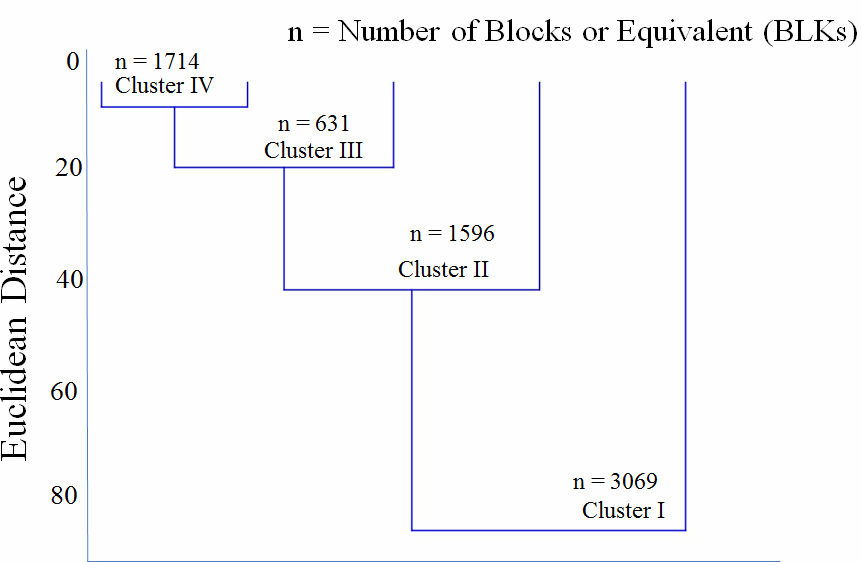


# S2.4 Standard Error

We have estimated standard error for each of the analysis. Errors associated with NL_an_, SAN_an_, FC_an_ and AD_an_ were determined by estimating the standard deviation.

Error estimation were calculated by following equation,

Standard error (SE) = δ/√n (1)

Where, δ = standard deviation and n = number of observation References:

1. Hausman, J. A., and D. L. McFadden. Specification tests for the multinomial logit model. Econometrica 52:1219–1240 (1984).
2. Lessig VP. Comparing cluster analyses with cophenetic correlation. J. Mark. Res. 9(1):82–84. 10.2307/3149615 (1972)
